# Cytological Changes and Immunocytochemistry Expression of P53 in Oral Mucosa Among Waterpipe Users in the Kingdom of Saudi Arabia

**DOI:** 10.7759/cureus.31190

**Published:** 2022-11-07

**Authors:** Faris M Elmahdi, Rawan O Alsebaee, Mayar M Ballaji, Ahmad M Alharbi, Majed E Alhejaili, Huda S Altamimi, Majed J Abu Altaher, Maysan B Ballaji, Ahmed S Aljohani, Ahmed A Alahmdi, Renad N Alsaedi, Mawaddah S Aeq, Ahmed S Alahmadi, Abdulrahman F Alraddadi, Yahya F Jamous

**Affiliations:** 1 College of Medicine, Department of Histology, Al Rayyan Colleges, Madinah, SAU; 2 College of Medicine, Al Rayyan Colleges, Madinah, SAU; 3 Molecular Pharmacology, Vaccines and Bioprocessing Center, King Abdulaziz City for Science and Technology (KACST), Riyadh, SAU

**Keywords:** ksa, water pipe, oral mucosa, p53 expression, cytological changes

## Abstract

Objective

In this study, we aimed to assess cytological changes and p53 expression in oral mucosa among waterpipe users in the Kingdom of Saudi Arabia (KSA).

Methodology

A case-control study was conducted in KSA from January to October 2022. Two cytologic oral smear samples each were taken from 500 volunteers; 300 were waterpipe users (case) while 200 did not use a waterpipe (control). They were then stained using the Papanicolaou staining procedure and immunocytochemical method to show the expression of P53.

Results

The interpretation of the Papanicolaou staining outcomes showed the presence of four results with different proportions: inflammation, infection, atypia, and keratinization. Cytological inflammation was identified among 77/300 (25.6%) waterpipe smokers, which was higher than that among non-users (12/200, 6%). The reverse cytological infection and atypia were also higher in waterpipe smokers compared with controls (9% vs. 4.5% and 4.3% vs. 0.5%, respectively), and keratinization was detected only in waterpipe users (3.6%) compared with controls. Waterpipe users had higher p53 protein expression than non-users.

Conclusion

Using a waterpipe is an effective way to change the oral mucosa. In atypia and keratinization, there was high p53 expression. These results could indicate that p53 is involved in both the change from normal to cancerous cells and the growth of new cells, but the presence or absence of p53 staining could not be used to predict the outcome of potentially cancerous oral mucosal lesions.

## Introduction

Tobacco use is directly responsible for approximately 85% of all cases of oral cancer. One major risk factor for oral cavity cancer is the use of tobacco products, including cigarettes and waterpipes. Based on the calculated toxicity, a single session of hookah smoking is equivalent to anywhere from one to 50 cigarettes [1]. Smoking a hookah poses the same health risks as smoking cigarettes. In contrast, the risk of developing oral cancer due to smoking can be reduced if patients quit smoking at an early age. Oral cancer rates worldwide have been shown to correspond with the prevalence of tobacco use [2].

Many Saudis, especially young people, have fallen ill from smoking in recent years [3]. According to the alarming findings of a recent study, 40.8% of male secondary school students in northern Saudi Arabia are active smokers. Cigarettes account for 67.3% of tobacco use, followed by shisha at 22.4%. About 39.8% of Saudi Arabian adolescents surveyed said they smoked cigarettes daily, with 29.6% saying they smoked fewer than five cigarettes per day [4].

Although shisha is a tobacco-related product, its potentially harmful effects on health have not been analyzed to the same extent as those of cigarette smoking. There is a pressing need to study and document the negative effects of waterpipe smoking on oral health, particularly among young people who are increasingly falling prey to the habit, so that it can be regulated like other forms of tobacco. Additionally, young people who smoke shisha need to be properly warned about the dangers of engaging in this habit. Hence, the goal of this study was to look into the effects of shisha smokers on the mouths of smokers and compare it with people who do not smoke.

Several previous studies have used p53 immunohistochemical (IHC) markers as proliferative cell markers for oral cancer; this marker has been shown to be reliable as a proliferative marker because it is expressed during all active phases of the cell cycle (G1, S, G2, and mitosis) except the G0 phase [5].

Even though tobacco consumption via waterpipes is linked with cigarette-like unfavorable health consequences and its use is on the rise among the young, it remains a vastly underexplored field of study in many aspects. In light of this, this research aimed to assess the impact of smoking a waterpipe on the cytomorphological and immunocytochemistry pattern of the oral epithelium.

## Materials and methods

For this case-control study, which was conducted from January to October 2022, 500 people in good health were chosen at random. Participants in this study were all male and Saudi residents. Cases included shisha smokers (n=300) and controls included non-smokers (n=200). All study participants had two buccal smears taken from them (employing all necessary safety precautions and sample adequacy measures). With their ages ranging from 18 to 65 years old and a mean age of 23 years, both patients and controls appeared to be in good health. Each person had their buccal smear and sputum samples taken. A carefully prepared questionnaire was used to gather personal information and other demographic details.

Sample collection

Buccal Smear

A wooden tongue depressor was used to collect exfoliative cells from the oral mucosa, which includes the tongue's dorsum and both cheeks. The cells were then directly spread on two clean glass slides and were fixed instantly in 95% ethyl alcohol while still moist. Buccal smears were sent to the histopathology lab at the Rayyan College of Medicine in Saudi Arabia for staining and diagnosis.

Papanicolaou’s stain

Following fixation in ethanol, smears were hydrated for two minutes in a series of ethanol solutions, decreasing in strength from 95% to 70% in distilled water. Smears were stained with Harris hematoxylin for five minutes to visualize the nuclei, rinsed in distilled water to remove any excess stain, differentiated in 0.5% aqueous hydrochloric acid for 10 seconds to remove any excess stain particles, and finally rinsed in distilled water to halt the decolorization process. The smears were then dehydrated in ethanol ranging from 70% to twice the concentration of 95% for two minutes at a time, after being dyed blue in alkaline water for four seconds. Following a two-minute incubation in Papanicolaou Orange G6 solution, a 95% ethanol rinse, and a three-minute incubation in Papanicolaou EA50 staining solution, smears were examined for cytoplasmic staining. The smears were then dehydrated in 95% absolute ethanol, washed in xylene, and mounted on Dibutylphthalate Polystyrene Xylene (DPX) [6].

Immunocytochemical method

The smear was rinsed with phosphate-buffered saline (PBS) three times for three minutes each. Each slice was treated with 0.3% hydrogen peroxide in methanol for 15 minutes to suppress endogenous peroxidase activity, followed by three PBS rinses. These antibodies (Abs) were utilized: sections were incubated with a primary mouse monoclonal p53 antibody (Gene Tech Company Limited, Shanghai, China) at a working dilution of 1/100 for 30 minutes at 37 °C; following two washes in PBS, sections were incubated with a secondary antibody, Chem Mate TM En Vision of+/HRP (Gene Tech Company Limited), at room temperature for 30 minutes; and finally, sections were washed three times in PBS. The immunoreactivity was detected using a 1/100 dilution of diaminobenzidine (DAB) (Gene Tech Company Limited) as the final chromogen for 10 minutes, followed by a three-minute wash in DW. Finally, sections were counterstained with hematoxylin for three minutes, rinsed for five minutes in running tap water, dehydrated in alcoholic solutions, cleaned in xylene, and mounted with DPX. In only one instance, P53 expression was identified as a distinct brown cytoplasmic staining in epithelial cells and nuclei [7].

Cytological assessment

All Pap-stained smears from cases and controls were analyzed for cytopathological abnormalities. We looked for signs of inflammation, infection, atypia, and keratinization. Cytological alterations were characterized by features including bi- or multinucleation, uneven.

Ethical consent

Before the specimen was obtained, each participant was requested to complete an ethical consent form in writing. The King Abdulaziz City for Science and Technology (KACST) Ethical Committee created and approved the informed ethical consent form (Grant no. AT-87.34).

## Results

This study examined 500 healthy adults between the ages of 18 and 64 years; the mean age of the subjects was 34 years. Most shisha smokers (90/300, 30%) were in the age range of 31-40 years, followed by 41-50 years (77/300, 25.7%). Most of the non-smokers (90/200, 47%) were in the age group of 18-30 years, followed by 31-40 years (45/200, 12%), as shown in Table 1.

**Table 1:** Distribution of the study population by age

Age (years)	Case (n=300)		Control (n=200)		P-value
	N	%	N	%	
18-30	55	18.3	94	47	0.5
31-40	90	30	45	12	
41-50	77	25.7	32	16	
51-60	54	18	25	12.5	
>60	24	8	4	2	

The interpretation of the Papanicolaou staining outcomes showed four results with different proportions: inflammation, infection, atypia, and keratinization. Cytological inflammation was identified among 77/300 (25.6%) waterpipe smokers, which was higher than that among non-users (12/200, 6%). The reverse cytological infection and atypia were also higher in waterpipe smokers compared with controls (9% vs. 4.5% and 4.3% vs. 0.5%, respectively), and keratinization was detected only in waterpipe users (3.6%) compared with control groups. The rate of abnormal results was 42.7% in waterpipe users and 11% in non-users (p=0.041), as indicated in Table 2.

**Table 2:** Frequency of cytopathological changes among the study population

Papanicolaou staining result	Case (n=300)		Control (n=200)		P-value
	N	%	N	%	
All diagnosed	128	42.7	22	11	0.041
Atypia	13	4.3	1	0.5	
Inflammation	77	25.6	12	6	
Infection	27	9	9	4.5	
Keratinization	11	3.6	0	0	

**Figure 1:**
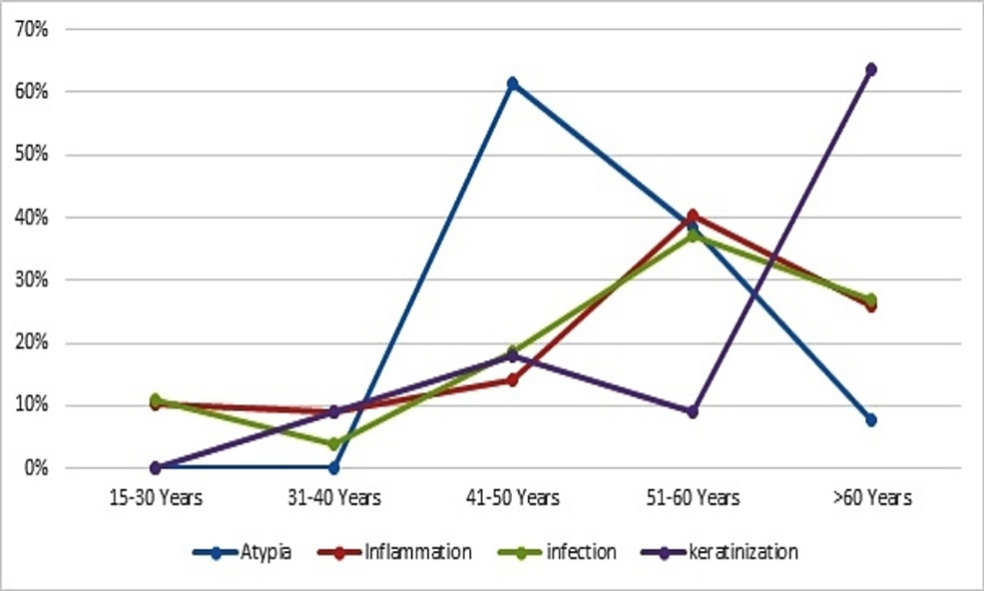
Distribution of the cytological changes in waterpipe users by age

About eight of 13 (61.5%) of the atypical cytologic cases were seen in the age groups of 41-50 years. Most of the cases of inflammation and infection were seen in the age range of 51-60 years, accounting for 31/77 (40.2%) and 10/27 (37%) subjects, respectively. Most of the cases of keratinization were seen in the age group >60 years, representing 7/11 (63.6%) subjects, as indicated in Figure 1.

Most cases of cytologic atypia (8/13, 61.5%) had a duration of 16-20 years, followed by ≥21 years (3/13, 23%), and 11-15 years (2/13, 15%). Most of the inflammatory cases (50/77, 64.9%) had a duration of 11-15 years. Most of the cases of infection (10/27, 37%) were associated with a duration ≥21 years. Most of the keratinization cases had durations of 6-10 years, 16-20 years, and ≥21 years, constituting 3/11 (27%) for all, as shown in Table 3.

**Table 3:** Cytological changes according to the duration of waterpipe use

		≤5 years	6-10 years	11-15 years	16-20 years	≥21 years
Atypia	0 (0%)		0 (0%)	2 (15.8%)	8 (61.5%)	3 (23.1%)
Inflammation	10 (12.9%)		5 (6.5%)	50 (64.9%)	5 (6.5%)	7 (9%)
Infection	4 (14.8%)		1 (3.7%)	7 (25.9%)	5 (18.5%)	10 (37%)
keratinization	0 (0%)		3 (27%)	2 (18.1%)	3 (27%)	3 (27%)

Waterpipe use resulted in high p53 expression in 5% of cases and low p53 expression in 280 (95% of the cases), whereas the control group had low p53 expression for all subjects, as shown in Table 4.

**Table 4:** P53 expression among the study population

P53 immunohistochemistry	Case (n=300)		Control (n=200)		P-value
	N	%	N	%	
High p53 (%)	20	10	0	0	0.045
Low p53 (%)	280	90	200	100	

**Figure 2:**
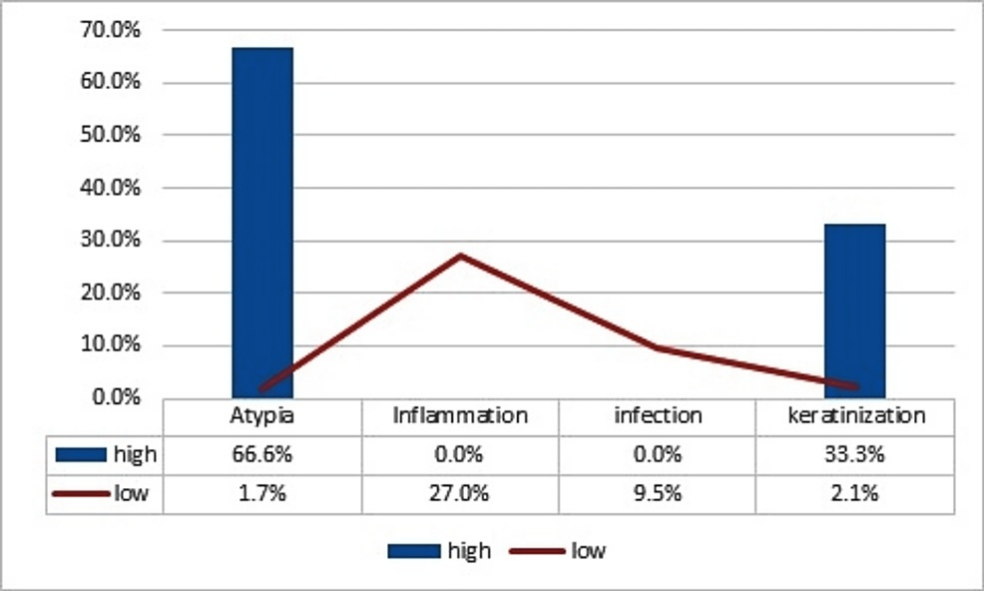
Relationship between cytological changes and p53 expression in waterpipe users

Most cases of cytologic atypia and keratinization showed a high expression of p53 at 66.6% and 33.3%, respectively. While infection and inflammation showed a low expression, as shown in Figure 2.

**Figure 3:**
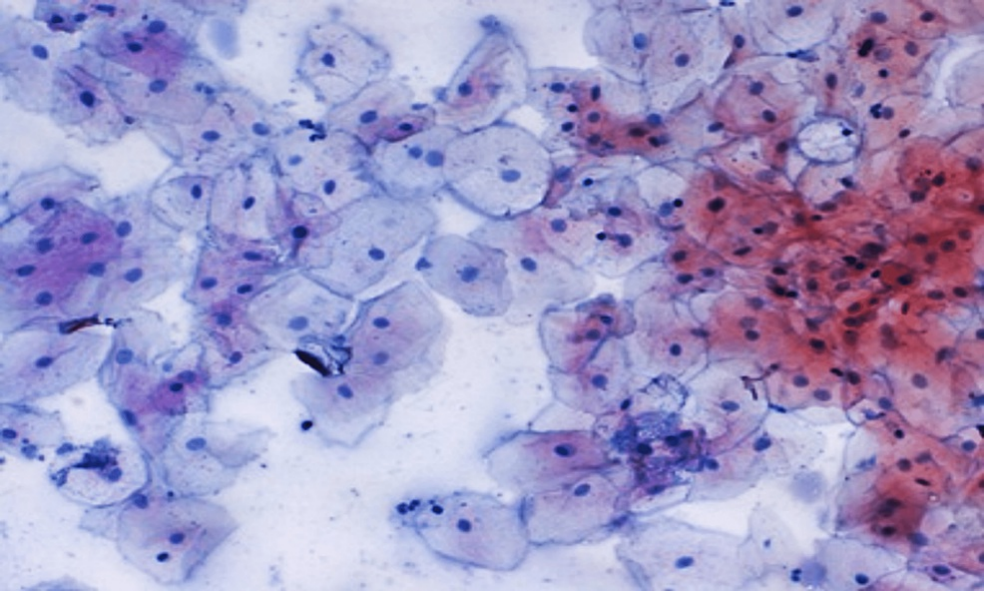
Photomicrograph of a buccal cell containing a high expression of P53 (immunocytochemistry 400x)

**Figure 4:**
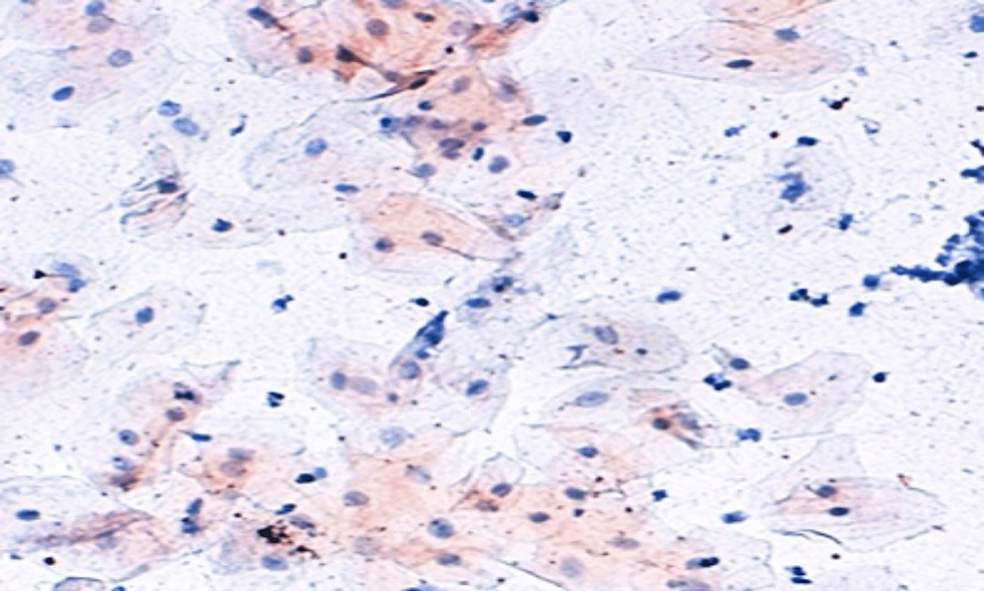
Photomicrograph of a buccal cell containing a low expression of p53 (immunocytochemistry 400x)

A photomicrograph of a buccal cell containing a high expression of P53 is depicted in Figure 3, while a photomicrograph of a buccal cell containing a low expression of p53 is shown in Figure 4.

**Figure 5:**
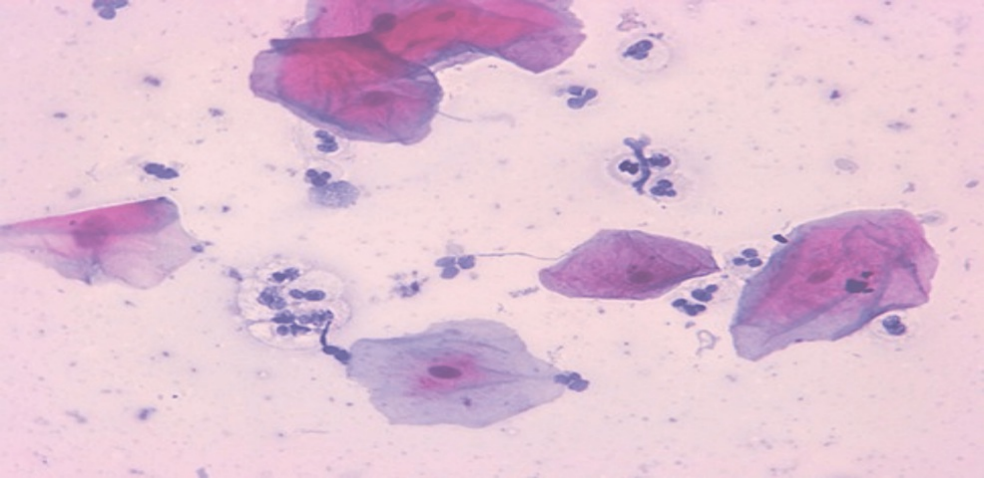
Photomicrograph of a buccal cell containing inflammatory cells (Pap stain 400x)

**Figure 6:**
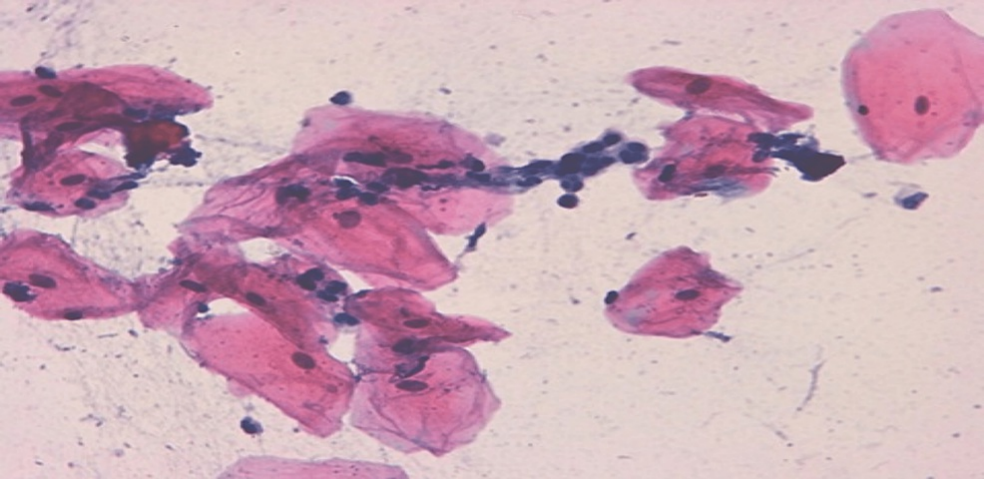
Photomicrograph of a buccal cell containing atypia (Pap stain 400x)

Figure 5 shows a photomicrograph of a buccal cell containing inflammatory cells, while a photomicrograph of a buccal cell containing atypia is illustrated in Figure 6.

## Discussion

Shisha smoking has been associated with various oral side effects, including discoloration of teeth and restorations, a diminished sense of smell and taste, periodontitis, peri-implantitis, precancerous lesions, etc. [6]. Some studies have found that the risk of cancer is comparable for smokers and waterpipe users, while others have found that waterpipe use is more dangerous than smoking [7].

This study showed the results in terms of the relationship between smoking shisha and changes in the oral epithelium. These findings demonstrated a strong link between shisha use and the risk of oral epithelial atypical changes, which can lead to precancerous or cancerous changes in the mouth. Similar studies in Africa have shown higher rates, such as the study by Agabeldour et al. (2016) from Sudan. Their study involving 70 people who used waterpipes regularly showed that 47.1% had cytomorphologically unusual changes, while none of the controls did. This slight difference from our findings could be attributed to their sample size being smaller than ours [8].

Studies have shown that smokers and people who use waterpipes are more likely to have changes in their oral mucosal epithelia that could lead to cancer. In a cross-sectional analysis, cytologic smear samples were collected from the oral mucosa, the tongue's lateral surface, and the mouth floor (right) of 40 waterpipe users and 40 non-users. The study discovered that smoking and waterpipe use both resulted in quantifiable cytometric alterations in the oral mucosa. However, smoking had a more pronounced effect on cytometric changes than using a waterpipe [9]. In addition, another study undertaken in Jeddah, Saudi Arabia, found that cigarette smoking was the most prevalent form of tobacco use (65.6%), followed by waterpipe use (38.1%). By way of conventional clinical examination, it was discovered that tobacco users had a high prevalence of soft tissue lesions (88.8%) and that almost 50% of them had hairy tongues, smoker's melanosis, stomatitis nicotine, frictional keratosis, fissured tongues, gingival or periodontal inflammation, and leukoedema. Only 0.5% of the patients had suspicious (premalignant) lesions, with smokeless keratosis (6.3%), leukoplakia (2.3%), erythroplakia (0.7%), oral submucous fibrosis (0.5%), and lichenoid lesions (0.4%) being the most prevalent. The high incidence of oral mucosal soft tissue lesions (88.8%) may be attributable to the irritant action of tobacco on oral tissues [10]. However, in this investigation, the frequency of cytological alterations was greater than that reported in a previous study. This could be because cigar smokers and people who drink toombak, both of which can cause oral epithelial atypical changes, are represented in the group.

Furthermore, it was reported that the deteriorative effects increased with a longer duration of exposure. Similar findings have been reported previously with respect to toombak dipping and smoking cigarettes [11]. Also, since tobacco tends to restrict peripheral blood vessels, it negatively affects the mouth wound's healing process. Carbon monoxide and other products of tobacco burning can restrict blood flow in the capillaries. According to a clinical trial, a single cigarette can decrease peripheral blood velocity by 40% per hour [12].

We looked into normal oral mucosa p53 expression in shisha smokers because p53 changes that happen early in the process of oral cavity carcinogenesis are linked to smoking. In the present investigation, in line with numerous other global studies, we discovered a link between smoking and P53 positivity. The fact that p53 was found in the mucosa of smokers' samples could indicate that smoking shisha causes early mucosal changes that lead to oral squamous cell carcinoma (OSCC) [13]. However, smoking is associated with p53 mutations in OSCC [14] and premalignant lesions; Lazarus et al. [15] have proposed that p53 mutation may manifest as a very early event in the evolution of oral cavity tumors and found that non-smokers' premalignant lesions lack p53 mutations. Also, the expression of p53 above the basal layer in the epithelium is seen as an early stage of oral carcinogenesis and a sign of a growing carcinoma, even if morphological tissue changes have already happened [16]. This research also indicated that shisha smoking is more prevalent among adults ≥40 years of age, which may represent a future threat to a significant segment of the community. Hence, efforts to make smoking less of a problem and the use of screening programs are seen as critical for controlling smoking in the future. According to a study, waterpipe use is related to considerable oral epithelial alterations among Sudanese users. People who smoke shisha should take part in regular screening programs to find out if they have conditions that could lead to cancer or are already cancerous.

This study has a few limitations, one of them being our exclusion criteria. Moreover, due to its design, this study could not determine the causes of anomalous results, such as p53 expressions and the association between them and the kind, duration, and age of smokeless tobacco use. To address the limitations of this work, additional research employing molecular techniques to determine the mutation of the p53 gene is required.

## Conclusions

Using a waterpipe is an effective way to change the oral mucosa. In atypia and keratinization, there was high p53 expression. These results suggest that p53 is involved in both the change from normal to cancerous cells and the growth of new cells, but the presence or absence of p53 staining could not be used to predict the outcome of potentially cancerous oral mucosal lesions. More research needs to be conducted on how immunocytochemistry and cytology can be used to diagnose OSCC in people who use waterpipes.
